# *eGFP* Gene Integration in *HO*: A Metabolomic Impact?

**DOI:** 10.3390/microorganisms10040781

**Published:** 2022-04-06

**Authors:** Fanny Bordet, Rémy Romanet, Camille Eicher, Cosette Grandvalet, Géraldine Klein, Régis Gougeon, Anne Julien-Ortiz, Chloé Roullier-Gall, Hervé Alexandre

**Affiliations:** 1 Institut Agro Dijon, PAM UMR A 02.102, Institut Universitaire de la Vigne et du Vin (IUVV), Université Bourgogne Franche-Comté, Rue Claude Ladrey, BP 27877, CEDEX, 21000 Dijon, France; remy.romanet@u-bourgogne.fr (R.R.); camille.eicher@gmail.com (C.E.); cosette.grandvalet@agrosupdijon.fr (C.G.); geraldine.klein@u-bourgogne.fr (G.K.); regis.gougeon@u-bourgogne.fr (R.G.); chloe.roullier-gall@u-bourgogne.fr (C.R.-G.); rvalex@u-bourgogne.fr (H.A.); 2 Lallemand SAS, 19 Rue des Briquetiers, CEDEX, 31700 Blagnac, France; ajulien@lallemand.com; 3 DIVVA (Développement Innovation Vigne Vin Aliments) Platform/PAM UMR, Institut Universitaire de la Vigne et du Vin (IUVV), Rue Claude Ladrey, BP 27877, CEDEX, 21000 Dijon, France

**Keywords:** *S. cerevisiae*, eGFP, *HO* gene, CRISPR-Cas9 technology, metabolomics

## Abstract

Integrating fluorescent genes including e*GFP* in the yeast genome is common practice for various applications, including cell visualization and population monitoring. The transformation of a commercial *S. cerevisiae* strain by integrating a cassette including a gene encoding an EGFP protein in the *HO* gene was carried out using CRISPR-Cas9 technology. Although this type of integration is often used and described as neutral at the phenotypic level of the cell, we have highlighted that under alcoholic fermentation (in a Chardonnay must), it has an impact on the exometabolome. We observed 41 and 82 unique biomarkers for the S3 and S3GFP strains, respectively, as well as 28 biomarkers whose concentrations varied significantly between the wild-type and the modified strains. These biomarkers were mainly found to correspond to peptides. Despite similar phenotypic growth and fermentation parameters, high-resolution mass spectrometry allowed us to demonstrate, for the first time, that the peptidome is modified when integrating this cassette in the *HO* gene.

## 1. Introduction

Fluorescent labeling or cell tracing has previously been used extensively to track a multi-dimensional system [1,2,3,4,5]. The discrimination of subpopulations is then possible via fluorescence analysis and quantification. Different fluorescent proteins for the fluorescent tagging of *Saccharomyces cerevisiae* are used to perform cell dynamics measurements in culture. The Green Fluorescent Protein (GFP), discovered in 1962 by Shimomura [6] and derived from the jellyfish *Aequorea victoria,* is currently the best known. This protein is composed of 11 beta-sheets and an alpha-helix including the chromophore and loops between the sheets. It undergoes cyclization and is then oxidized to form the chromophore p-hydroxybenzylidene-imidazolidinone, which will become mature after a cycle of protonation–deprotonation. This EGFP protein initially showed two absorption wavelengths at 395 and 477 nm and an emission peak at 509 nm. Different variants of this protein have been developed, including the EGFPS65T protein, making it possible to simplify the absorption spectrum and reduce chromophore formation time [7,8]. The use of this protein has different applications, such as the use of the gene coding it as a reporter gene and the use of the protein as a molecular or cellular marker [9].

The selection of the target gene for *eGFP* gene integration remains crucial. Thus, the *HO* gene of the yeast *S. cerevisiae*, which is responsible for the diploidization of haploid cells, has proven to be a tool of choice. Indeed, this gene, which codes for an endonuclease, configures the mating type and, thus, initiates transposition between the a and alpha types of haploid cells [10,11,12], is inactive in diploid cells. Since most oenological yeasts are diploid, there is no transcription of this gene due to the inhibition of the gene by the a1 and alpha2 products. Therefore, the inactivation of this gene through the integration of a gene coding for a cell marking protein, such as *EGFP*, should not impact the cell phenotype.

However, in all studies focusing on the monitoring of cell dynamics, it is essential to confirm the absence of impact of this integration in order to exhaustively evaluate the behavior of the subpopulations present. Few phenotypic changes contributing to the common use of tagged yeast have been observed or reported. In 1994, Chalfie et al. reported that cells grew normally even in the presence of the gene coding for EGFP and suggested that EGFP was a good candidate for cell separation with fluorescently activated cells. They also considered that *EGFP* could be used as a vital marker to assess cell growth [13]. Nevertheless, some studies have noted changes, including protein localization. Furthermore, it was found that when EGFP was used as a fusion protein, depending on the target protein, the localization of the labeling (N- or C-end tagging) could induce changes in the expression of *EGFP*, including the localization of fluorophore expression [14]. These results were confirmed by targeting the proteins essential for yeast growth. Thus, the authors were able to demonstrate that, in some cases, this modification of the subcellular localization of protein expression was associated with a modification of cell fitness [15].

Most studies focus on the phenotypic impact and certain metabolic features of the integration of genes coding for an EGFP, but few have focused on the impact on the overall metabolism of the cell. In this study, different approaches were combined to determine the impact at different scales of the integration of a gene into the *HO* gene of a commercial diploid *S. cerevisiae* strain. The metabolomic approach was employed, for the first time, to characterize the effect of this transformation.

## 2. Materials and Methods

### 2.1. Microbial Strains

The *Saccharomyces cerevisiae* parental strain used in the *eGFP* integration by the CRISPR-Cas9 system was a commercial strain coded S3 (Lallemand Inc, Montreal, Canada). *E. coli* DH5α was used as plasmid vector for S3GFP construction. S3 and *E. coli* DH5α were stored at −80 °C, respectively, in YPD liquid medium (0.5% (*w*/*v*) yeast extract (Biokar, Beauvais, France), 1% (w/v) Bacto-peptone (Biokar), 2% (*w*/*v*) D-glucose (Prolabo, Fontenay-sous-Bois, France), and 0.02% (*w*/*v*) chloramphenicol (Sigma, Saint-Louis, MI, USA)) containing 20% *v*/*v* glycerol and LB liquid medium (0.5% (*w*/*v*) yeast extract (Biokar, Beauvais, France), 1% (*w*/*v*) Bacto-peptone (Biokar), 1% (*w*/*v*) NaCl (Prolabo, Fontenay-sous-Bois, France)) containing 20% *v*/*v* glycerol.

### 2.2. Constructions of Yeast Strains Labeled with EGFP Using CRISPR-Cas9 Tool

EGFP *S. cerevisiae* strains were constructed using the CRISPR-Cas9 tool (Figure S1) carrying the pCE vector derived from the pRCC-K vector [16]. The sgRNA was designed to target the *S. cerevisiae HO* gene using gRNA designer (ATUM) (Table 1). The plasmid pCE was constructed by inserting the gRNA-oligotarget1-*HO* downstream from the SNR52 promoter by digesting pRCC-K with *BamH*I and using the NEBuilder HiFi DNA assembly Cloning kit (New England Biolabs, Ipswich, USA) according to the manufacturer’s recommendations. *E. coli* DH5α recombinant strains were selected on LB plates supplemented with ampicillin (100 µg mL^−1^). The insertion of sgRNA in pRCC-K was checked via plasmid DNA extraction using NucleoSpin Plasmid (Macherey-Nagel, Düren, Germany), and then through the Sanger sequencing of the insertion region with Genewiz^®^ (Leipzig, Germany) using the primer V1-AS-F (Table 1).

A donor DNA fragment containing the *eGFP* gene and the nourseothricin resistance cassette was amplified from the pFA6a-TEF2Pr-*eGFP*-ADH1-NATMX4 plasmid [17] using primers (Fluo-F-*HO* and Fluo-R-*HO*, Table 1) and Taq polymerase (ROCHE) according to the manufacturer’s instructions. Both primers contained floating tails at 5′ corresponding to the insertion sequence of the *HO* gene, enabling homolog recombination in the *S. cerevisiae* S3 genome.

The LiAc transformation method was used to transfer pCE and donor DNA fragments into *S. cerevisiae*. Competent yeast cells were prepared as follows: *S. cerevisiae* cells were grown in YPD medium at 28 °C under shaking until reaching an optical density at 600 nm (OD_600nm_) of 2. Cells (100 UDO (unit of optical density)) were harvested via centrifugation (3730× *g* for 5 min at room temperature (RT)) and washed in 10 mM Tris pH 7.5. Yeasts were centrifuged for 3730× *g* 5 min at RT and resuspended in LiAc Buffer (0.1 M lithium acetate, 10 mM Tris, pH 7.5) and incubated for 40 min at 20 °C. Competent yeast cells were centrifuged at 2000× *g* for 5 min at RT and resuspended in 2.25 mL of LiAc Buffer.

pCE plasmid (500 ng), *eGFP*–nourseothricin fragment (1 µg) and salmon testes ssDNA (1 g, Sigma) were mixed with the competent cells (final volume 100 µL) and incubated for 10 min at 20 °C without agitation. Then, 300 µL of 50% PEG 4000 was added before incubating them for 10 additional minutes at 20 °C. Yeast cells were then heat-shocked at 42 °C for 15 min. PEG was removed by centrifugation (2000× *g* for 4 min at RT), and transformed cells were resuspended in 500 µL YPD and incubated at 30 °C overnight with gentle shaking. *S. cerevisiae* recombinant strains were selected on YPD plates supplemented with nourseothricin (75 µg mL^−1^, Sigma). Homologous recombination was verified using PCR amplification on yeast recombinant strains as follows. Chromosomal DNA was extracted from yeast colonies using the InstaGene™ Matrix kit (Bio Rad, Hercules, CA, USA and DNA was amplified using GoTaq^®^ DNA polymerase (Promega) according to the manufacturer’s instructions. The *eGFP*–nourseothricin fragment was amplified using primers Verif*GFP*-F and Verif*GFP*-R (Table 1) and recombination into the *HO* gene was checked using primers V1*HO*-F V2*HO*-R and V1*HO*-F Verif*GFP*-R (Table 1).

### 2.3. Fermentation Process

Yeast strains were grown on YPD plates (YPD broth with 2% *w*/*v* agar) at 28 °C from the stock at −80 °C. From each YPD plate, one isolated colony was inoculated in 15 mL of YPD medium, and the liquid cultures were incubated at 28°C for 24 h. These precultures were diluted at a concentration of 0.1% (*v*/*v*) in 150 mL of YPD medium (0.5% (*w*/*v*) yeast extract, 1% (*w*/*v*) Bacto-peptone, 2% (*w*/*v*) glucose, and 0.02% (*w*/*v*) chloramphenicol) in 250 mL Erlenmeyer flasks. After incubation at 28 °C with stirring (150 rpm) for 18 h, a second culture in 150 mL filtered Chardonnay must (0.22 µM membrane, ClearLine, Dutscher, Dumath, France) was performed in 250 mL Erlenmeyer flasks at 28 °C in static mode for 18 h. These cultures were used to inoculate pasteurized Chardonnay must containing 226.6 g.L^−1^ glucose/fructose, pH 3.92, and 343.1 g.L^−1^ total assimilable nitrogen, at 1 × 10^6^ viable cells.mL^−1^. The fermentations were performed in 2 L bottles containing 1 L of inoculated must covered with sterile cotton wool. For each strain, assays were conducted in three biological replicates at 20 °C without stirring. The end of fermentation was considered to be the total depletion of sugars.

### 2.4. Flow Cytometry Analysis

#### 2.4.1. Yeast Viability

Yeast viability in preculture and during fermentation was determined via flow cytometry. The fluorochrome used was Propidium Iodide (PI) (Invitrogen, Molecular Probes, ThermoFisher Scientific, Illkrich, France) dissolved in filtered milliQ water at a concentration of 0.1 mg mL^−1^ [4]. Here, 1 µL of PI was added to 100 µL of diluted yeast suspension in PBS buffer (ThermoFisher Scientific, Illkrich, France). Samples were incubated in the dark 15 min before measurement.

#### 2.4.2. Flow Cytometry Settings

Flow cytometry was performed with a BD Accuri C6 flow cytometer, and the resulting data were processed using BD Accuri C6 software. A 488 nm wavelength argon laser was used to excite the cells (autofluorescence) and dye.

For each sample, 20 µL of each sample was analyzed at 34 µLmin ^−1^. The FSH threshold used was 80,000. PI and GFP fluorescence were measured on the FL3-H long pass filter (675 nm) and on the FL1-H channel (533/530 nm), respectively. The fluorescence intensity of the relevant channel was plotted as Side SCatter light (SSC) and analyzed. Cell viability was determined by subtracting the PI-labeled dead population from the total population. Gates were drawn to delimit the wild type and fluorescent yeast populations. Daily sampling of samples was carried out.

### 2.5. Two-Photon Microscopy

Two-photon imaging microscopy was performed to obtain 2D representations of the two *Saccharomyces cerevisiae* strains. The images were collected on a Nikon A1-MP scanning microscope equipped with a Plan APO IR × 60 objective (NA, 1.27; Water Immersion, Nikon, Tokyo, Japan) at a scanning speed of one frame per second. An IR laser (Chameleon, Coherent, Palo Alto, CA, USA) was used to provide excitation at 820 nm. Fluorescence emission was collected on two detection channels: FF01-492/SP (400–492 nm) and FF03-525/50 (500–550 nm) (Semrock, New York, USA). The images provided in this article were obtained by merging these two detection channels.

Samples of the culture were collected and centrifuged at 8000× *g* for 5 min at 20 °C. The culture supernatant was removed, and the pellet was resuspended in PBS buffer (137 mM NaCl, 2.7 mM KCl, and 11.9 mM phosphate, pH 7.2) (ThermoFisher Scientific, Illkrich, France). Five microliters of cell suspension were placed between a glass slide and a coverslip for observation.

### 2.6. Analytical Method

#### 2.6.1. Enological Analysis

To determine the sugar concentration and ethanol degree, the samples were centrifuged at 8000× *g* for 5 min at 4 °C and were monitored daily using FTIR (Fourier Transformed InfraRed) spectroscopy (OenoFOSS ^TM^, FOSS, Hilleroed, Denmark). Daily sampling of all the samples was carried out.

#### 2.6.2. UHPLC-Q-ToF-MS Analysis

Analyses were performed using ultra-high-pressure liquid chromatography (Dionex Ultimate 3000, Thermo Fisher Scientific, Waltham, MA, USA) coupled with a MaXis plus MQ ESI-Q-ToF mass spectrometer (Bruker, Bremen, Germany). Non-polar compounds were analyzed in reverse phase using an Acquity BEH C18 1.7% m, 100 × 2.1 mm column (Waters, Guyancourt, France). The mobile phase was ultrapure water from a Milli-Q system (Merck, Darmstadt, Germany) + 0.1% (*v*/*v*) of formic acid (MS grade) (Acros Organic, Morris Plains, NJ, USA) for eluent A, and 95% (*v*/*v*) acetonitrile (MS grade) (Biosolve, Dieuze, France) + 0.1 (*v*/*v*) of formic acid for eluent B. The temperature of elution was 40 °C using the gradient: 0-1.10 min 5% (*v*/*v*) of eluent B and 95% of eluent B at 6.40 min. The flow was 400 µLmin^−1^. The nebulizer pressure was 2 bar and 10 l.min^−1^ for the nitrogen dry gas flow. Ionization was performed in electrospray in positive or negative ion mode. The ion transfer parameters were 500 V for the end plate offset and 4500 V for the capillary voltage. Before each batch analysis, the mass spectrometer was calibrated using undiluted Tuning Mix (Agilent Technologies, Santa Clara, CA, USA) in enhanced quadratic mode (errors <0.5 ppm). The mass range was between 100 and 1000 *m*/*z*. To verify the stability of the UHPLC-Q-TOF-MS system, quality controls (a mix of all the samples analyzed in the batch analysis) were analyzed at the beginning, the end, and for every 10 samples during the batch analysis. All the samples were analyzed randomly. The injection of the calibrant ESI-L Low-concentration Tuning Mix, diluted 4 times at the beginning of each run, allowed us to recalibrate the spectrum.

DataAnalysis (v. 4,3, Bruker, Mannheim, Germany) was used for the internal mass recalibration of the spectrum and extraction of molecular features (couple *m*/*z*, retention time). Spectral background noise was removed before feature extraction (S/N > 30, absolute intensity >1000). A homemade R script was used for feature alignment with *m*/*z* and a retention time tolerance lower than 10 ppm and 0.3 min, respectively. MATLAB (R2015a) was used for statistical analysis (PCA, *t*-test, FDR control) and data visualization. Isolated significant features were annotated using the online database Metlin, KEGG, and Oligonet online tools [18,19,20]. Van Krevelen plots were produced using the elementary formula obtained by annotation.

## 3. Results and Discussion

### 3.1. Fluorescence Emission of Recombinant Yeast GFP

The *S. cerevisiae* commercial strain coded S3 was genetically modified by integrating the *eGFP* gene into the chromosomal *HO* gene using the CRISPR-Cas9 tool. The correct integration of *eGFP* into both *HO* alleles of the S3 diploid strain has been checked by PCR amplifications using three couples of primers (Table 1) as described in Material and Methods (data not shown).

**Figure 1:**
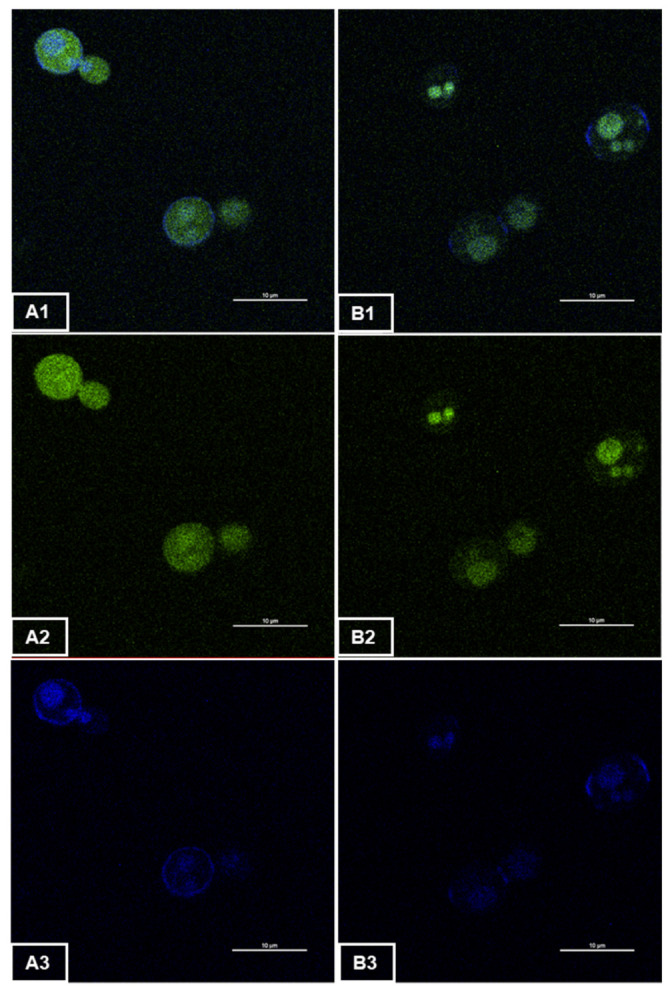
Two-photon microscopy observation of (**A**) the modified strain S3GFP and (**B**) the wild-type strain S3. The images (**A2**,**B2**) were obtained with channel FF01-492/SP (400–492 nm) and (**A3**,**B3**) with channel FF03-525/50 (500–550 nm). The images (**A1**,**B1**) provided were obtained by merging these two detection channels.

Two-photon microscopy was used to confirm the expression of the *eGFP* gene in *Saccharomyces cerevisiae* and visualize EGFP localization in cells (Figure 1). We observed considerable homogeneity of the intensity and distribution of the fluorescence emitted by the cells throughout the sample. The images presented above are, therefore, representative of each of the samples studied. The wild-type strains (Figure 1(B1,B2)) exhibited a green auto-fluorescence, which could coincide with intracellular compartments, such as the vacuole or nuclei, as previously described [21,22,23]. Thus, a blue fluorescence was detected on another channel (Figure 1(A3,B3)) when observing the samples corresponding to both the wild-type strain and the modified strain. This signal seemed to be emitted at the region of the cell wall and was more intense at the bud junctions and budding scars. We could also observe a considerable difference in green fluorescence emissions between modified (Figure 1(A1,A2)) and wild-type cells (Figure 1(B1,B2)). Indeed, the auto-fluorescence emitted by the wild-type strain appeared to be less intense than for the modified strain. This confirmed the correct integration of the gene coding for the EGFP protein, as confirmed by the verification PCR (data not shown) as well as the production of the chromophore in its mature form. The latter did not appear to be localized in a defined cellular compartment. The fluorescence was diffused throughout the cell as expected. Therefore, the protein was apparently expressed in the cytoplasm. The use of two-photon microscopy allowed us to confirm that the fluorescence observed was inside the cell and not only localized at the yeast walls.

### 3.2. Growth and Fermentation Kinetics

**Figure 2:**
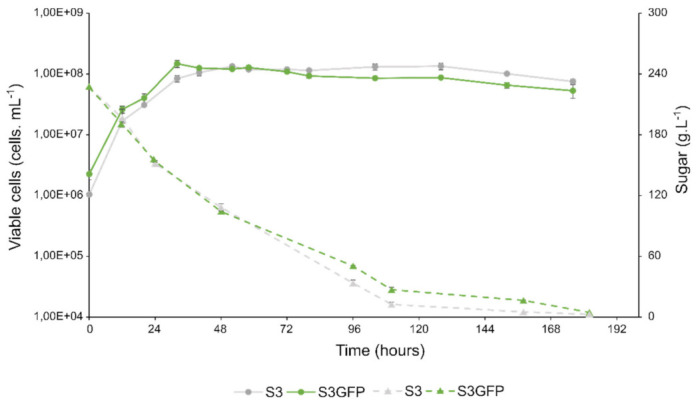
Fermentation profile comparison in Chardonnay must. Cell viability and sugar consumption were monitored for the *Saccharomyces cerevisiae* wild-type strain S3 (in grey) and the recombinant strain S3GFP (in green). Solid curves represent viable cell populations and dotted curves represent sugar concentrations. For each strain, the experiments were performed in triplicate and the error bars represent the confidence interval.

We investigated the impact of the integration of the e*GFP* gene into the *HO* gene on various phenotypic parameters usually used to evaluate the performance of oenological yeast strains undergoing alcoholic fermentation. The growth of the two yeast strains in Chardonnay must as well as the fermentation kinetics were monitored via flow cytometry and infrared spectroscopy (IRTF) every day until the complete depletion of sugars occurred (Figure 2).

The two strains S3 (wild-type strain) and S3GFP (recombinant strain) showed similar growth kinetics.

The maximum growth rate (µMax), the number of generations, and the maximum population (K) were determined for each strain (Table 2). No significant differences were observed for the different parameters evaluated, which validates the absence of the phenotypic impact of *eGFP* integration into the *HO* gene relative to those growth kinetics parameters [24]. The maximum growth rates (µMax) of strains S3 and S3GFP were 1.37 × 10^−1^ ± 5 × 10^−3^ h^−1^ and 1.39 × 10^−1^ ± 4 × 10^−3^ h^−1^, respectively. Both strains also reached equivalent maximum populations (KS3 =1.42 × 10^8^ ± 2 × 10^6^ viable cells mL^−1^, KS3GFP =1.50 × 10^8^ ± 1 × 10^7^ viable cells mL^−1^). As described previously [25], it can be concluded that the interruption of the *HO* gene seems not modify the growth capacity of the modified strain and its fermentative kinetics.

On the other hand, in order to evaluate the capacity to degrade sugars, the time necessary to degrade half of the sugars available in the medium (T50) and to reach the maximum speed of consumption of sugars (Vmax) were defined (Table 2). It was observed that the maximum rate was not significantly different, i.e., at 1.80 ± 0.32 gL^−1^h^−1^ and 2.06 ± 0.12 gL^−1^h^−1^ for the S3 and S3GFP strains, respectively. Similarly, the T50 did not differ significantly (T50_S3_ = 45.3 ± 0.9 h and T50_S3GFP_ = 43.3 ± 0.4 h). The results also revealed that the end of fermentation occurred at the same time for both strains (264 h). The fermentative performance of the wild-type strain was maintained despite the integration of the gene encoding the EGFP.

Thus, from the point of view of fermentation and growth kinetics, the *HO* gene seemed to be a neutral target for *Saccharomyces cerevisiae* transformation. This absence of impact on the phenotype could be conducted under different environmental conditions in order to avoid a possible protein burden phenomenon. Indeed, this phenomenon was described by Kafri et al. in 2016 [26], when considering sensitive competitive growth experiments, showed that depending on the nutrient or cellular resources, the protein burden was modulated.

### 3.3. Metabolomic Pattern

UPLC-qToF-MS (metabolomic) analysis of post-alcoholic fermentation wines from the two strains studied was performed to evaluate the impact of the yeast genetic modification on its metabolome. Here, 2235 features were detected in both positive and negative ionization modes.

Statistical analysis (*t*-test, FDR control (q < 0.05) and log_2_ (fold change) >|0.5|) allowed us to isolate 199 features that significatively discriminated between S3 and S3GFP; therefore, less than 10% of the total number of ions were included. Putative annotation was assigned for each significant extracted feature using an online database (Metlin, KEGG) and the online tool Oligonet. Thus, 151 features were annotated at level 3 according to Schymanski et al. 2014 [27] (Table S1). Therefore, only annotated features were considered to ascertain the impact of the transformation of the *Saccharomyces cerevisiae* strain, and considered as biomarkers.

**Figure 3:**
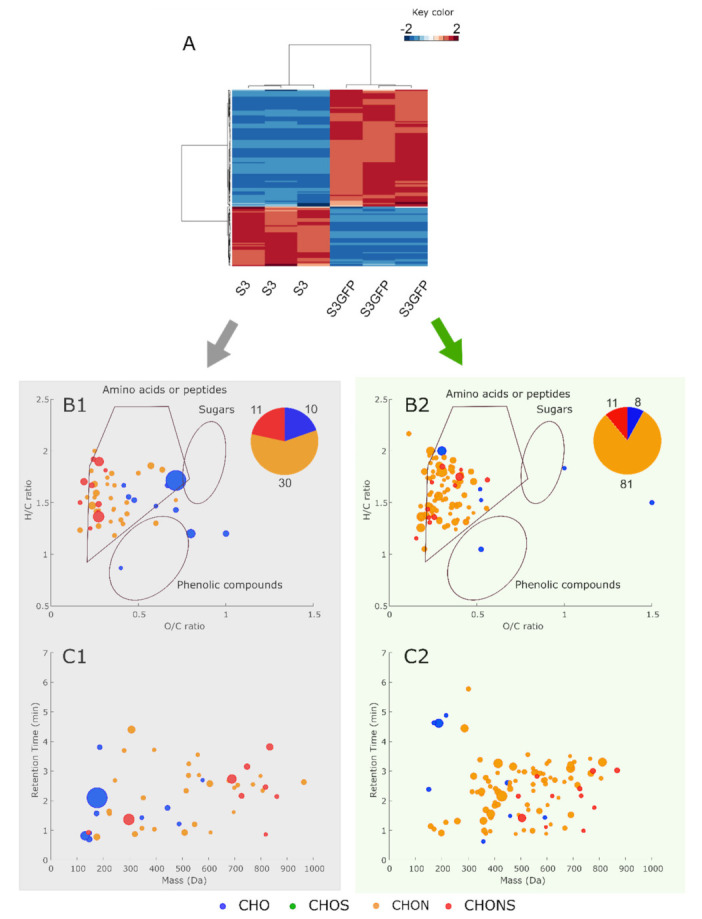
(**A**) Heatmap based on the 151 significatively different annotated compounds for S3 and S3GFP in Chardonnay must. (**B1**) and (**B2**) represent the van Krevelen diagrams for the compounds that are significatively more present in S3 and S3GFP (in red on the heatmap), respectively. (**C1**) and (**C2**) represent retention time versus mass (Da) for the compounds significatively more present in S3 and S3GFP, respectively. The area of bubbles corresponds to the square root of detected area divided by 10.

A large number of these biomarkers appeared to be unique to each modality. Indeed, 41 and 80 biomarkers were specific to the S3 and S3GFP strains, respectively, while 28 were detected at significantly different concentrations in both strains (Figure 3A). The largest number of biomarkers was found for the modified strain S3GFP. This number of biomarkers highlights the impact of strain transformation.

To go further, we were interested in the nature of the modifications occurring with the genetic modification of the yeast. Van Krevelen diagrams were established for each strain using the biomarkers most present (Figure 3B). The van Krevelen diagrams were based on their O/C and H/C ratios and associated with the areas of compound families according to the composition of the compounds. Considering the latter, we observed a low diversity of composition between the biomarkers of the two strains. It was mostly CHON compounds that were expressed for both strains, which was confirmed by the associated pie chart that represents the number of markers classified according to their molecular formulas. This highest number of CHON compounds was located where peptides were expected in the van Krevelen diagrams. The associated retention time versus mass plots (Figure 3C) confirmed the predicted family of compounds to be peptides because of the relatively low retention times ((<4 min) using reverse phase column = polar compounds).

However, it was found that the proportion of CHON compounds (which are peptide-like) was significantly higher for the S3GFP strain, with 81% of all biomarkers compared to 59% for the S3 strain. Additionally, we observed a high composition diversity within the compounds in CHO and CHONS that were found in the same number in both strains.

The metabolomic fingerprint of the S3 wild-type strain was modified by the integration of the *eGFP* gene within the *HO* gene in the modified strain S3GFP. Furthermore, this powerful technique allowed us to distinguish the same strain differing only by the integration of a gene within its genome.

We were additionally focused on the hypothetical identity of biomarkers in each strain and the metabolic pathways impacted by the genetic transformation. Among the possible biomarker metabolites of the wild-type S3 strain, propylmalate (Level 3) (m = 176.0683 Da, C_7_H_12_O_5_, RT = 2.1 min) was significantly more present. It has been reported that this compound is an intermediate of leucine biosynthesis [28]. Moreover, it seemed that the main potential annotated compounds for strain S3 were intermediates of amino acid biosynthetic pathways, with the exception of tyrosine, in contrast to most of the annotated markers specific to the S3GFP strain with 82 peptides. In addition, the other biomarkers identified could be involved in the metabolism of ascorbate and aldarate. These latter aspects could contribute towards the hypothesis regarding the response to oxidative stress by the modified yeast S3GFP. Indeed, it has been reported that EGFP can induce oxidative stress [29]. Additionally, tyrosine, peptides, and ascorbate are described as participating in an antioxidant effect [30,31]. However, we were not able to determine if this was related to the cassette insertion or the disruption of the *HO* gene or a polar effect, as very few studies have focused on this. Although some teams have assayed some key metabolites in their research, this remains undetermined. Furthermore, Gui et al. in 2021 [24] reported that the location of the integration of the target gene encoding EGFP was a key factor in its transcription. Indeed, the study of 250 strains of *S. cerevisiae*, in which a gene coding for the EGFP protein was integrated at different locations in the genome, revealed competition for transcriptional responses and resources. This could be due to the recruitment of transcriptional resources by the highly integrated promoter, making them unavailable to nearby genes. Conversely, ricochet effects leading to transcriptional synergy have also been suggested by this team. Moreover, protein folding disorders have been demonstrated during fusion processing with a fluorescent protein [32], and the fusion construct may form a network of interacting proteins leading to aggregation [9]. It has also been described that protein overexpression could lead to an overload of cellular resources for translation, folding, or degradation [33,34]. This overexpression could be generated during this integration or associated with the use of a strong promoter, such as pTEF2.

Finally, the peptides extracted as biomarkers from the S3GFP-transformed strain could be peptides derived from the protein.

## 4. Conclusions

This work reported the metabolomic impact of *eGFP* gene integration in the *HO* gene of *Saccharomyces cerevisiae*. Despite similar phenotypes, the production of a hundred new metabolites as well as the overexpression of some by the modified strain compared to the wild-type strain led to the conclusion that the integration of this *eGFP* gene at the chosen location is not neutral. Therefore, a variation of the exo-metabolome was observed, especially on the peptidome. The changes observed, which might be due to either the localization of the integrated cassette, the disruption of the *HO* gene, or the production of EGFP and its derivatives, remain to be studied. However, UHPLC-MS turned out to be useful to describe the impact of transformations on yeast metabolism. Although the changes observed do not seem to affect the phenotype, in our experimental conditions, it would be interesting to check for phenotypic differences in sub-optimal culture conditions.

## Figures and Tables

**Table 1:** Primers used in this study. sgRNA sequences targeting the *HO* gene have been underlined for gRNA-oligotarget1-*HO*. Floating tails of Fluo-F-*HO* and Flu-R-*HO* primers enabling recombination have been underlined.

Primer	Sequence (5′-3′) *
**gRNA-oligotarget1-*HO***	CTCCGCAGTGAAAGATAAATGATCAAATTGGGCATTACGCCCGA GTTTTAGAGCTAGAAATAGCAAGTTAAAATAAGG
**V1-AS-F**	GTAGTGCCCTCTTGGGCTA
**Fluo-F-*HO***	AAAAAGGCAAAAGACAAAGGCGAAAAATTGGGCATTACGCCGGTCGACGGATCCCCGGGTT
**Fluo-R-*HO***	CACATTTTATACACTCTGGTCCTTTAACTGGCAAACCTTCGTCGATGAATTCGAGCTCGTT
**Verif*GFP*-F**	ACGGCGTCGTACAAGAGAAC
**Verif*GFP*-R**	TACATAACCTTCGGGCATGG
**V1*HO*-F**	CATACGACTGTAATGTTGCT
**V2-*HO*-R**	AAACTGTAAGATTCCGCCAC

* sgRNA sequences targeting the *HO* gene have been underlined for gRNA-oligotarget1-*HO*. Floating tails of Fluo-F-*HO* and Flu-R-*HO* primers enabling recombination have been underlined.

**Table 2:** Growth and fermentation kinetics parameters for strains S3 and S3GFP in Chardonnay must.

Strain	Maximum Population Growth Rate: µ Max (h^−1^)		Generation Number		Maximum Population: K (Viable Cells.mL^−1^)		Maximum Sugar Consumption Rate: Vmax (gL^−1^h^−1^)		Time to T50 of Sugar Degradation (Hours)	
**S3**	1.37 × 10^−1^ ± 5 × 10^−3^	}NS	4.75 ± 0.18	}NS	1.42 × 10^8^ ± 2 × 10^6^	}NS	1.80 ± 0.32	}NS	45.3 ± 0.9	}NS
**S3GFP**	1.39 × 10^−1^ ± 4 × 10^−3^		4.54 ± 0.16		1.50 × 10^8^ ± 1 × 10^7^		2.06 ± 0.12		43.3 ± 0.4	

Values correspond to the average of three biological replicates ± standard deviation. Statistical analysis was performed between both strain fermentations (t-test, α = 0.05) (NS: not significant).

## Supplementary Materials

The following supporting information can be downloaded at: https://www.mdpi.com/article/10.3390/microorganisms10040781/s1, Figure S1: Scheme illustrating *HO* gene deletion and insertion of DNA donor by CRISPR/Cas9 tool for EGFP *Saccharomyces cerevisiae* tagging.; Table S1: List of annotated biomarkers.
