# Supplementary material for: eGFP Gene Integration in HO: A Metabolomic Impact?
Source: Microorganisms. 2022 Apr 6;10(4):781. doi: 10.3390/microorganisms10040781 (PMC9032140; doi:10.3390/microorganisms10040781)
Supplement: Supplementary file 1 [file microorganisms-10-00781-s001.zip › microorganisms-1632576-supplementary.pdf]

Supplementary data

Microorganisms- 1632576

eGFP gene integration in *HO*: a metabolomic impact?

Fanny Bordet*[1,2], Rémy Romanet[1], Camille Eicher[1], Cosette Grandvalet[1], Géraldine Klein[1], Régis Gougeon[1,3], Anne Julien-Ortiz[2], Chloé Roullier-Gall[1] and Hervé Alexandre[1]

[1] Université Bourgogne Franche-Comté, Institut Agro Dijon, PAM UMR A 02.102, F-21000 Dijon, France- Institut Universitaire de la Vigne et du Vin (IUVV), Rue Claude Ladrey, BP 27877, CEDEX, bor det.fanny@gmail.com (F.B.); remy.romanet@u-bourgogne.fr (R.R.), camille.eicher@gmail.com (C.E.), Chloe.Roullier-Gall@u-bourgogne.fr (C.R.- G.), regis.gougeon@u-bourgogne.fr (R.G.);rvalex@u-bourgogne.fr (H.A.)

[2] Lallemand SAS, 19 Rue des Briquetiers, CEDEX, 31700 Blagnac, France, ajulien@lallemand.com (A.J.-O.)

[3] DIVVA (Développement Innovation Vigne Vin Aliments) Platform/PAM UMR, IUVV, Rue Claude Ladrey, BP 27877, CEDEX, 21078 Dijon, France

* Correspondence: bordet.fanny@gmail.com

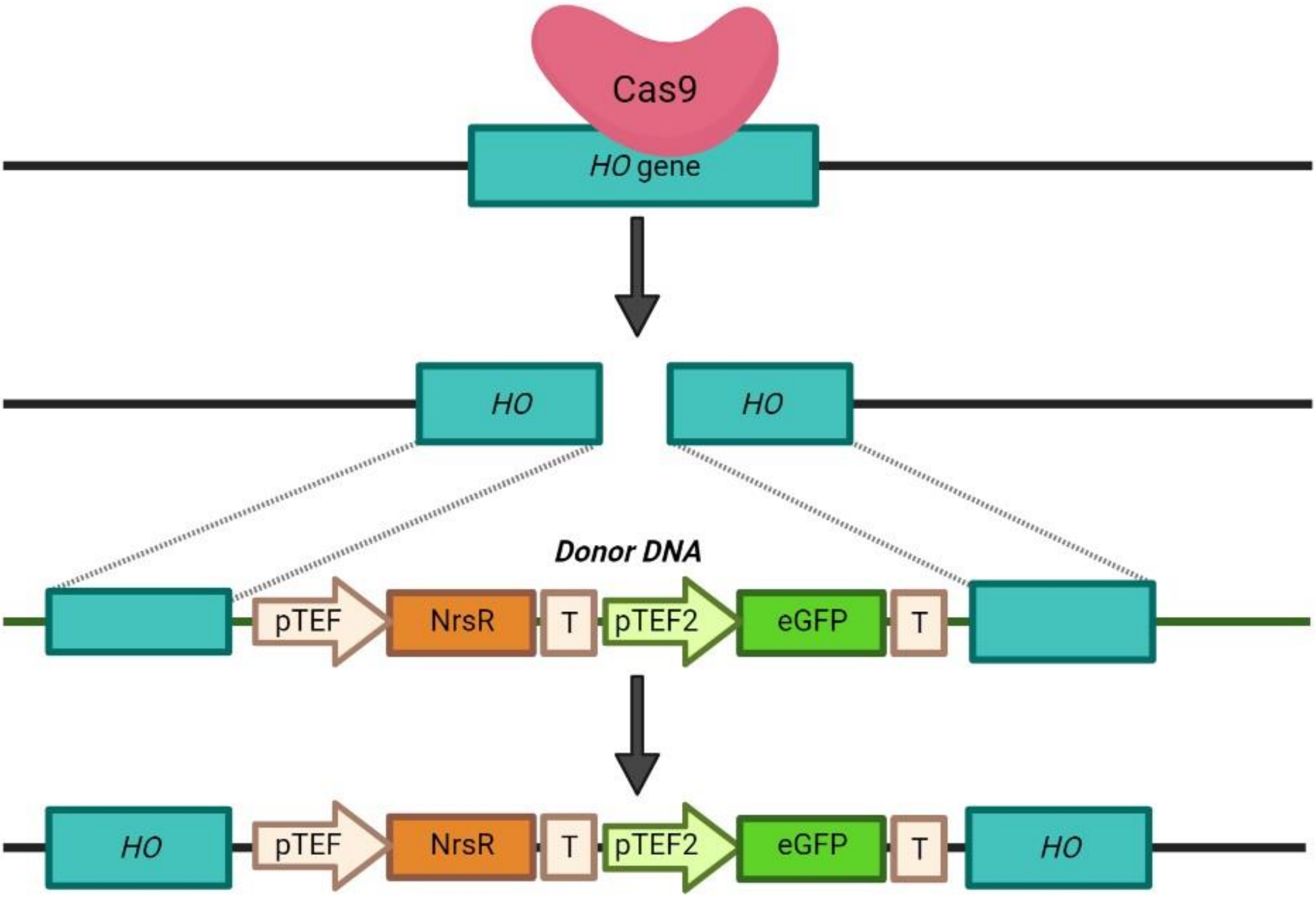

Supplemental Figure S1: Scheme illustrating *HO* gene deletion and insertion of DNA donor by CRISPR/Cas9 tool for EGFP *Saccharomyces cerevisiae* tagging.

Supplemental Table S1: List of annotated biomarkers

m/z	RT (min)	Annotation error (ppm)	Molecular Formula	Database annotation	Condition
130,02658	0,81726	0,24317	C5H6O4	'(E)-Glutaconate; trans-Glutaconate; trans-Glutaconic acid; Glutaconic acid; 2,5-Dioxopentanoate; 2-Oxoglutarate semialdehyde; 2-Methylmaleate; Citraconate; Citraconic acid; Methylmaleic acid; 4,5-Dioxopentanoate; 4-Oxoglutarate semialdehyde; Acetylpyruvate; Acetylpyruvic acid; 2,4-Dioxopentanoate; Itaconate; Itaconic acid; Methylenesuccinic acid; Mesaconate; 2-Methylfumarate; Mesaconic acid; Methylfumaric acid; (E)-2-Methyl-2-butenedioic acid'	S3
143,04056	0,93368	-0,50961	C6H9NOS	'5-(2-Hydroxyethyl)-4-methylthiazole; 4-Methyl-5-(2''-hydroxyethyl)-thiazole; 4-Methyl-5-(2-hydroxyethyl)thiazole; HET'	S3
146,02138	0,70405	0,98971	C5H6O5	'2-Hydroxyparaconate; 2-Oxoglutarate; Oxoglutaric acid; 2-Ketoglutaric acid; alpha-Ketoglutaric acid; 5-Hydroxy-2,4-dioxopentanoate; Dehydro-D-arabinono-1,4-lactone; (5R)-3,4-Dihydroxy-5-(hydroxymethyl)furan-2(5H)-one; Methyloxaloacetate; Oxaloacetate 4-methyl ester'	S3
146,05773	0,91210	1,22554	C6H10O4	'(R)-3-Hydroxy-3-methyl-2-oxopentanoate; (R)-4-Dehydropantoate; (S)-2-Aceto-2-hydroxybutanoate; (S)-2-Hydroxy-2-ethyl-3-oxobutanoate; 2-Aceto-2-hydroxybutanoate; 2-Dehydropantoate; 3-Hydroxy-3-methyl-2-oxopentanoic acid; 3-Hydroxy-3-methyl-2-oxopentanoate; 3-Hydroxy-5-oxohexanoate; 4-Hydroxy-2-oxohexanoic acid; 4-Hydroxy-2-oxohexanoate; Adipate; Adipic acid; Hexanedioate; Hexan-1,6-dicarboxylate; Mevaldate; Mevaldic acid'	S3
174,05258	1,57235	1,38261	C7H10O5	'(2S)-2-Isopropyl-3-oxosuccinate; 3-Carboxy-4-methyl-2-oxopentanoate; 2-Oxo-4-methyl-3-carboxypentanoate; 2-Methyl-3-oxoadipate; 2-Oxopimelate; alpha-Ketopimelate; 2-Oxoheptanedionic acid; 4-Methyl-3-oxoadipate; Demethyl-4-deoxygadusol; 2,3,5-Trihydroxy-5-(hydroxymethyl)cyclohex-2-en-1-one; Shikimate; Shikimic acid; 3,4,5-Trihydroxy-1-cyclohexenecarboxylic acid; Valienone'	S3

175,08464	0,77807	-1,05653	C7H13NO4	'Calystegin B2; Calystegine B2'	S3
176,06828	2,10096	1,10980	C7H12O5	'(2R,3S)-3-Isopropylmalate; 3-Isopropylmalate; 3-Carboxy-2-hydroxy-4-methylpentanoate; 2-D-threo-Hydroxy-3-carboxy-isocaproate; (R)-2-(n-Propyl)-malate; 2-Propylmalate; 2-Propylmalic acid; Malic acid, 2-propyl-; 3-Propylmalate; Validone; alpha-Isopropylmalate; (2S)-2-Isopropylmalate; (2S)-2-Hydroxy-2-isopropylsuccinic acid; 2-Isopropylmalic acid; 3-Carboxy-3-hydroxy-4-methylpentanoate; 3-Carboxy-3-hydroxyisocaproate'	S3
186,08891	3,80776	1,58373	C9H14O4	'cis-2-Carboxycyclohexyl-acetic acid'	S3
222,10066	1,63675	0,9657;-0,97028	C11H14O3N2	GF'N5-Phenyl-L-glutamine; gamma-Glutamylanilide'	S3
223,08425	1,56778	0,93238	C11H13NO4	'Bendiocarb; 2,2-Dimethyl-1,3-benzodioxol-4-ol methylcarbamate; Dioxacarb'	S3
244,17851	2,68909	0,74076;-0,74076	C12H24O3N2	LL'Leucyl-leucine; N-(6-Aminohexanoyl)-6-aminohexanoate'	S3
279,14682	3,69833	0,85601	C15H21NO4	'Metalaxyl; Metalaxyl-M'	S3
297,08978	1,36952	-0,73411	C11H15N5O3S	'5''-Methylthioadenosine; Methylthioadenosine; S-Methyl-5''-thioadenosine; 5-Methylthioadenosine; 5''-Deoxy-5''-(methylthio)adenosine; Thiomethyladenosine; MTA; 5''-Deoxy-5''-(methylsulfanyl)adenosine'	S3
320,13300	0,87386	0,6124;-0,60615	C11H20O7N4	Q /GASS /N /GGST /AGSS /GGST'2-[(L-Alanin-3-ylcarbamoyl)methyl]-3-(2-aminoethylcarbamoyl)-2-hydroxypropanoate'	S3
346,12622	1,43052	0,46327	C15H22O9	'Aucubin; Deutzioside'	S3
347,16913	1,07133	0,36102	C14H25O7N3	DLT /ELS /ETV	S3
352,13691	2,09822	3,89881	C15H20N4O6	GN /GGY /GGGY	S3
393,22594	3,71744	1,10251;4,50259	C20H31O5N3	LVY'Oxidized Cypridina luciferin'	S3
394,14906	1,04000	0,54066	C17H22O7N4	DFN /GG /DFGG	S3
444,19965	1,75883	2,78028	C21H32O10	'Mitoxantrone'	S3
488,26205	1,22029	2,94104	C24H40O10	LPPY	S3
509,33476	0,92737	4,30985	C28H47NO7	HKLL	S3
515,29528	2,33800	0,44829	C23H41O8N5	DGLLV /ADLVV /EGLVV /AAELL /AEVVV /LLPSS /LPSTV /PTTVV	S3
524,21074	2,85625	2,11277	C23H32N4O10	DDLY /DEVY /EEFT	S3
525,35347	3,24733	1,59152	C26H47O6N5	ALLLP /LPVVV	S3
547,28017	1,20200	2.46596;1.27644;-1,278269	C30H37N5O5	ELMR /ACKLN /GG /GKMN /GGV /CGKLQ /GA /ACKQ /GAV /AAKMQ /GA; FPVW'Ergosine'	S3
559,33622	3,55529	1,37540	C29H45O6N5	AFLLP /FPVVV	S3
564,25469	2,86375	0,54305	C25H36O9N6	DFGLN /GG /ADFN /GGV /DFGQ /GAV /EFGN /GGV /AAEFQ /GA /SSSVW /FPQ /GASS /ASTTW /FN /GGPST /APQ /GASY /GTTTW /AN /GGPTY /GPQ /GATY	S3

578,14236	2,69538	4,14628;0,11910	C30H26O12	CCCHN /GG'Epicatechin-(4beta->8)-ent-epicatechin; Procyanidin B2; Procyanidol B2; Procyanidin B4; Catechin-(4alpha->8)-epicatechin; Procyanidin B5; Procyanidol B5'	S3
605,33098	2,57938	1,55617	C27H43N9O7	CKLRS /CKRTV /AKMRT	S3
608,19248	0,93243	3,96812	C21H32O15N6	ACDDW /CDEGW	S3
676,29351	2,67051	0,92093	C28H40O10N10	DHRSY /DN /GGRSW	S3
689,38876	2,73605	1,92405	C29H55O8N9S1	FKLPW	S3
696,38071	1,61571	1,81982	C31H52O10N8	AHKRW	S3
698,39550	2,43755	2,71193	C31H54O10N8	DPRRR	S3
711,30794	2,53000	1,31751	C30H45O13N7	HHQ /GAQ /GAY /HN /GGQ /GAQ /GAW	S3
726,33100	2,16474	3,71173	C30H50O7N10S2	EEFFR /EPRYY	S3
746,39863	3,17684	2,20970	C32H58O10N8S1	EMRRR	S3
747,33702	3,14526	1,38118	C33H49O9N9S1	PPWWY	S3
747,33806	3,15323	0,01778	C33H49O9N9S1	PPWWY	S3
150,01630	2,38580	0,92747	C4H6O6	'(R,R)-Tartaric acid; (R,R)-Tartrate; L-Tartaric acid; Tartaric acid; Tartrate; 2,3-Dihydroxybutanedioic acid; (2R,3R)-Tartaric acid; (+)-Tartaric acid; (S,S)-Tartaric acid; (S,S)-Tartrate; D-Tartrate; D-Tartaric acid; (2S,3S)-Tartaric acid; (-)-Tartaric acid; meso-Tartaric acid; meso-Tartrate'	S3GFP
157,07368	1,14128	1,33236	C7H11NO3	'2-Hydroxyethylclavam; Hydroxyethylclavam; 3-Methylcrotonylglycine; Ethadione; Paramethadione'	S3GFP
170,10573	1,04614	-1,17146	C8H14N2O2	'Levetiracetam'	S3GFP
170,13067	4,63000	0,08187	C10H18O2	'(+)-Neomatatabiol; (1R,2R,4S)-Limonene-1,2-diol; (1R,2R,4S)-Menth-8-ene-1,2-diol; (4R,7S)-7-Isopropyl-4-methyloxepan-2-one; (4R,7S)-4-Methyl-7-(1-methylethyl)-2-Oxepanone; Mentholactone; (6E)-8-Hydroxygeraniol; 10-Hydroxygeraniol; 8-Hydroxygeraniol; (E)-3,7-Dimethylocta-1,6-diene-3,8-diol; (6E)-8-Hydroxylinalool; (S)-3-Acetyloctanal; 2-exo-Hydroxy-1,8-cineole; 6-endo-Hydroxycineole; 2-endo-Hydroxy-1,8-cineole; (1R,4S,6R)-1,3,3-Trimethyl-2-oxabicyclo[2.2.2]octan-6-ol; 7-Isopropyl-4-methyloxepan-2-one; 8-Methyl-6-nonenoic acid; Citronellate; Citronellic acid; 3,7-Dimethyl-6-octenoic acid; Limonene-1,2-diol; (1S,2S,4R)-Limonene-1,2-diol; (1S,2S,4R)-Menth-8-ene-1,2-diol; p-Menth-8-ene-1,2-diol; 1-Methyl-4-(1-methylethenyl)-1,2-cyclohexanediol; Menth-8-ene-1,2-diol'	S3GFP

188,14094	4,61678	1,60726	C10H20O3	'10-Hydroxydecanoic acid; 10-Hydroxydecanoate; 6-Hydroxy-3,7-dimethyloctanoate'	S3GFP
198,13698	0,91409	-0,74765	C10H18N2O2	'Slaframine; (-)-Slaframine'	S3GFP
215,07940	1,27073	-0,10285	C9H13NO5	'Succinylproline; Succinyl-L-proline'	S3GFP
216,17223	4,88313	1,44561	C12H24O3	'12-Hydroxydodecanoic acid; omega-Hydroxydodecanoic acid; 7-Hydroxydodecanoate'	S3GFP
259,15336	1,31439	0,57542	C11H21O4N3	Q /GAL /AGL /AAV	S3GFP
286,18960	4,44524	-1,18833	C14H26N2O4	'N-Acetyl-leucyl-leucine'	S3GFP
301,29780	5,77550	0,91604	C18H39NO2	'Sphinganine; Dihydrosphingosine; 2-Amino-1,3-dihydroxyoctadecane'	S3GFP
315,21564	2,30574	0,51570	C15H29O4N3	ALL /VVV	S3GFP
321,16958	2,83229	2,25115	C16H23O4N3	FGV	S3GFP
335,18414	2,29097	1,10839	C17H25O4N3	FGL /AFV	S3GFP
345,22667	3,38568	0,85793	C16H31O5N3	LLT	S3GFP
351,14293	2,24127	0,31766	C16H21O6N3	ADF /EFG	S3GFP
351,14314	2,31762	0,30146	C16H21O6N3	ADF /EFG	S3GFP
358,11115	0,62576	-0,07971	C12H22O12	'Cellobionate; Cellobionic acid; 4-O-beta-D-Glucopyranosyl-D-gluconate'	S3GFP
358,25830	1,32309	0,82408	C17H34O4N4	KLV	S3GFP
361,18486	0,99121	0,11461	C15H27O7N3	ELT	S3GFP
367,13791	1,69456	0,10959;4,40225	C16H21O7N3	DFS /ADY /EGY'Flumetover; N-Ethyl-N-methyl-4-(trifluoromethyl)-2-(3,4-dimethoxyphenyl)benzamide; EMTDB'	S3GFP
367,13959	0,90533	4,45243;0,15979	C16H21N3O7	DFS /ADY /EGY'Flumetover; N-Ethyl-N-methyl-4-(trifluoromethyl)-2-(3,4-dimethoxyphenyl)benzamide; EMTDB'	S3GFP
367,15426	2,78023	2,87172	C20H21N3O4	WY	S3GFP
377,15886	2,66071	0,47005;4,04753	C18H23O6N3	DFP'Imidaprilat'	S3GFP
384,23722	2,48484	0,13441	C18H32O5N4	GLPV /APVV	S3GFP
384,24871	0,97000	0,52868	C17H32O4N6	LPR	S3GFP
386,26407	1,44516	0,22331	C17H34O4N6	LRV	S3GFP
386,26454	1,55793	1,00269	C17H34O4N6	LRV	S3GFP
401,22908	1,83688	4,11081	C17H31N5O6	AKPS /GKPT /GLN /GGV /AN /GGVV /GQ /GAVV /AALQ /GA /GGGLV /AGGVV /AAAGL /AAAAV	S3GFP
402,21160	1,94300	0,37045	C17H30O7N4	DGLV /ADVV /EGVV /AAEL /LPSS /PSTV	S3GFP
404,16960	2,40931	0,03890	C19H24O6N4	AEW	S3GFP
408,16444	1,82955	0,14722	C18H24O7N4	DFQ /GA /EFN /GG /ADFG /EFGG	S3GFP
412,26862	2,94167	0,11077	C20H36O5N4	ALLP /PVVV	S3GFP
414,21207	2,29897	1,49191	C18H30O7N4	ADLP /EGLP /AEPV /PPTT	S3GFP
414,22706	3,26245	0,85614;0,85855	C22H30O4N4	LPW'Tentoxin'	S3GFP
414,24810	1,87196	0,62680	C19H34O6N4	LPSV /PTVV	S3GFP
428,26380	2,15881	0,73121	C20H36O6N4	LLPS /LPTV	S3GFP
450,11632	2,60474	-0,22998	C21H22O11	'2'',3,4,4'',6''-Peptahydroxychalcone 4''-O-glucoside; PHC 4''-O-glucoside; 2'',3,4,4'',6''-Pentahydroxychalcone 4''-O-beta-D-glucoside; Astilbin;	S3GFP

				Neoastilbin; (2S,3S)-Taxifolin 3-rhamnoside; Neocarthamin'	
457,32606	2,51786	0,79343	C22H43O5N5	KLVV	S3GFP
459,26981	2,61567	1,11772	C20H37O7N5	DKVV /AEKL /LLQ /GAS /LLN /GGT /LQ /GATV /AGLLS /GSVVV /AALSV /GGLLT /AGLTV /AATVV	S3GFP
460,20745	2,40226	0,90599;4,92616	C21H28O6N6	Q /GAQ /GAW /AFHS /FGHT /AAHY /AAN /GGW /AGQ /GAW /AAGGW'17,21-Dihydroxypregn-4-ene-3,11,20-trione 21-(hydrogensuccinate); 21-(3-Carboxy-1-oxopropoxy)-17-hydroxypregn-4-ene-3,11,20-trione; Cortisone 21-succinate; Albaspidin BB; Aspidin; alpha-Kosin'	S3GFP
471,24821	3,13440	0,08488	C24H33O5N5	GLPW /APVW	S3GFP
471,24862	3,15414	0,96391	C24H33O5N5	GLPW /APVW	S3GFP
480,30578	2,67089	0,50685	C23H40O5N6	HLLV	S3GFP
481,18089	0,88143	0,01188	C20H27O9N5	DFN /GGS /ADN /GGY /DGQ /GAY /EGN /GGY /DFGGS /ADGGY /EGGGY	S3GFP
491,21990	2,15510	0,68796	C23H33O5N5S1	ACLW /GMVW	S3GFP
492,22236	1,52318	0,69702	C23H32O8N4	DPVY /EFPT	S3GFP
493,25362	2,98765	0,05367	C23H35O7N5	AEFK /KPSY /FLQ /GAS /FLN /GGT /FQ /GATV /N /GGVVY /ALQ /GAY /AFGLS /PPPPS /AAFSV /FGGLT /AFGTV /GGVVY /AAGLY /AAAVY	S3GFP
505,22081	1,41841	0,33941	C20H35O8N5S1	DLMQ /GA /ELMN /GG /EMQ /GAV /CDGLV /ACDVV /ADGLM /AADMV /CEGVV /AACEL /EGGLM /AEGMV /CLPSS /CPSTV /AMPST /GMPTT	S3GFP
512,21119	1,00478	1,27817;2,97083	C22H32O10N4	ETTY; CFLM	S3GFP
514,31257	2,97652	2,08155	C23H42O7N6	AKLPS /GKLPT /AKPTV /GLLN /GGV /ALN /GGVV /GLQ /GAVV /AALLQ /GA /AQ /GAVVV	S3GFP
525,27986	1,86385	0,01391	C24H39O8N5	DPPVV /AELPP	S3GFP
527,31095	3,55822	0,33503	C28H41O5N5	LLPW	S3GFP
529,27466	1,69579	2,74496;0,22075	C23H39O9N5	AFHR; DLPSV /DPTVV /EPSVV /AELPT	S3GFP
545,28100	0,98824	2,32076;0,129345	C22H39O9N7	HHPR; EELR /DGKLN /GG /ADKN /GGV /DGKQ /GAV /EGKN /GGV /AAEKQ /GA /KPQ /GASS /KN /GGPST /LN /GGN /GGSV /N /GGQ /GASVV /ALQ /GAQ /GAS /N /GGN /GGTVV /ALN /GGQ /GAT /GLQ /GAQ /GAT /AQ /GAQ /GATV	S3GFP
547,30029	3,09344	0,56527	C27H41O7N5	FPSVV /AFLPT /GLPVY /APVVY	S3GFP
547,31341	2,55300	2,87221	C26H41N7O6	LPRY /KKSW /HPPVV	S3GFP
548,30690	2,33609	0,33986	C25H40O6N8	FLN /GGR /FQ /GARV /FGGLR /AFGRV /AHKPP	S3GFP
551,25950	2,94727	0,66294	C25H37O9N5	EEFK /ELQ /GAY /ADFLS /DFGLT /ADFTV /DGVVY /AADLY /EFGLS	S3GFP

				/AEFSV /EFGTV /AEGLY /AAEVY /PSSVY /FPSTT /APTTY	
559,27544	2,97829	0,01430	C26H37O7N7	EFHK /HLQ /GAY /LN /GGQ /GAW /Q /GAQ /GAVW /FGHLS /AFHSV /FGHTV /AGHLY /AAHVY /AGLN /GGW /AAN /GGVW /GGLQ /GAW /AGQ /GAVW	S3GFP
562,19244	2,82000	0,82969	C26H34O6N4S2	CFMY	S3GFP
567,25508	2,90825	0,53866;1,81653	C25H37O10N5	FHHQ /GA /AFGHH; EEKY /DFLSS /DFSTV /ADLSY /DGLTY /ADTVY /EEPPP /EFSSV /EGLSY /AESVY /AEFTT /EGTVY /PSTTY	S3GFP
571,26564	1,65650	2,49009	C28H37N5O8	DFKY /LN /GGYY /Q /GAVYY /DFPPP /AFFST /FGSVY /FFGTT /AAFTY /GGLYY /AGVYY	S3GFP
574,33284	2,92867	1,95879;0,36738	C25H46O9N6	FPRR; DKLLS /DKLTV /EKLSV /EKTVV /LLQ /GATT	S3GFP
586,40608	2,90867	1,16131	C28H54O7N6	KLLLT	S3GFP
587,32864	2,69034	0,9608;1,30561	C25H45O9N7	ARRW; DKKPT /EKKPS /DKLN /GGV /DKQ /GAVV /EKN /GGVV /AEKLQ /GA /LLQ /GAQ /GAS /LLN /GGQ /GAT /LQ /GAQ /GATV	S3GFP
592,27295	1,43302	2,00135;2,79322	C27H44O14	DGHHK /HHN /GGSV /AHHQ /GAT; KMMPS /CLLMN /GG /MMN /GGVV /CLMQ /GAV /ALMMQ /GA	S3GFP
593,30671	2,70975	1,05342	C28H43O9N5	DFLLS /DFLTV /ADLLY /DVVVY /EFLSV /EFTVV /EGLLY /AELVY /LPTTY	S3GFP
594,26495	2,57537	1,45090;0,00804	C26H38O10N6	KMMW; ADDFK /DEFGK /DFLN /GGS /DFQ /GASV /DFN /GGTV /ADLN /GGY /DGLQ /GAY /ADQ /GAVY /EFN /GGSV /AEFQ /GAT /EGLN /GGY /AEN /GGVY /EGQ /GAVY /STTTW /PQ /GASTY /N /GGPTTY	S3GFP
596,23151	1,11239	2,82936	C22H40N6O9S2	CDKMT /CEKMS /MMQ /GAST /MMN /GGTT	S3GFP
597,22877	0,88792	0,91394;0,53776	C25H35O12N5	DDSVY /DDFTT /DEFST /ADETY /EEFSS /AEESY /EEGTY; CFMPT /CCLPY /GMMPY	S3GFP
599,30647	3,25383	0,46706;4,94557	C29H41O7N7	RVYY /FHLPS /FHPTV /AHLPY /ALN /GGPW /GLPQ /GAW /APQ /GAVW'Delphinine'	S3GFP
602,30182	1,85500	0,95522	C24H42O10N8	DDRVV /ADELR /EEGLR /AEERV /EPRTT /DKLN /GGN /GG /DKN /GGQ /GAV /EKN /GGN /GGV /AEKQ /GAQ /GA /LQ /GAQ /GAQ /GAS /LN /GGQ /GAQ /GAT /Q /GAQ /GAQ /GATV	S3GFP
613,34298	2,78138	0,90021	C27H47O9N7	EKLPQ /GA	S3GFP
614,32690	1,20227	1,04090	C27H46O10N6	EEKLP	S3GFP
620,23099	2,16179	1,88795	C24H40O9N6S2	DMMPQ /GA /EMMN /GGP	S3GFP
630,32324	2,69905	0,87344;1,24609	C27H46O11N6	AFHRT /GHRVY /GN /GGRVW /AAQ /GARW /HHLPQ /GA; EELLQ /GA	S3GFP
644,24373	2,10267	0,73833	C29H36O11N6	DFN /GGSY /ADN /GGYY /DGQ /GAYY /EGN /GGYY	S3GFP

666,36946	2,63690	0,93020	C30H50O9N8	KKQ /GATY	S3GFP
668,32822	1,07522	0,01457	C32H44O8N8	AEFFR /FPRSY /APRYY /DHLVW /EHVVW /FFKN /GGN /GG'14-Deacetylnudicauline; Lycaconitine'	S3GFP
688,36397	3,50787	2,45787;-2,174845	C30H52O12N6	HLRTY /AEKRW /LQ /GARSW /LN /GGRTW /Q /GARTVW /HKKN /GGY /KKN /GGN /GGW'Callichiline'	S3GFP
690,31728	3,21235	1,94616	C27H46O13N8	EFHKM /HLMQ /GAY /LMN /GGQ /GAW /MQ /GAQ /GAVW	S3GFP
690,37099	3,09768	0,33596;1,32818	C32H50O9N8	CRRRT; EKKTW	S3GFP
691,31804	2,76087	1,44726;0,48527;0,76885	C31H45O11N7	HHHVY /HHN /GGVW; EEKTW; KMMPW	S3GFP
691,35637	1,52564	1,35627	C33H45O6N11	HPPRW	S3GFP
704,38614	3,31304	0,38739	C33H52O9N8	MRRRS	S3GFP
716,37093	2,53077	1,23171	C30H52O12N8	AERRW /HKN /GGRY /KN /GGN /GGRW	S3GFP
725,26485	2,40673	1,13210;2,32753	C25H43O14N9S1	DEHYY /DDQ /GAWY /DEN /GGWY; CMTWW	S3GFP
739,31447	0,98765	0,98804	C39H45O6N7S1	CFVWW /AFMWW	S3GFP
764,40642	3,41978	2,94505927295406;2,31653963966511	C35H56O11N8	DRRRY; FRRTW	S3GFP
767,40869	2,92534	4,16084;0,67508	C36H53O8N11	FFLRW'Rimocidine'	S3GFP
775,42599	3,00688	2,00707	C33H61O10N9S1	KMRRW	S3GFP
811,38874	3,30462	1,16458	C39H49O7N13	HQ /GARWW	S3GFP
867,36989	3,02750	0,60355	C39H53O10N11S1	Q /GAWWWY	S3GFP
